# Draft Genome Sequence of Deoxynivalenol-Degrading Actinomycete Nocardioides sp. Strain LS1, Isolated from Wheat Leaves in Japan

**DOI:** 10.1128/MRA.01650-18

**Published:** 2019-03-07

**Authors:** Hiroyuki Morimura, Kazuma Uesaka, Michihiro Ito, Shigenobu Yoshida, Motoo Koitabashi, Seiya Tsushima, Ikuo Sato

**Affiliations:** a Graduate School of Bioagricultural Sciences, Nagoya University, Chikusa, Nagoya, Japan; b Tropical Biosphere Research Center, University of the Ryukyus, Nishihara, Okinawa, Japan; c Central Region Agricultural Research Center, National Agriculture and Food Research Organization (NARO), Tsukuba, Ibaraki, Japan; d Department of Molecular Microbiology, Tokyo University of Agriculture, Setagaya, Tokyo, Japan; University of Arizona

## Abstract

Actinomycete Nocardioides sp. strain LS1, isolated from wheat leaf, is a bacterium that degrades and assimilates the mycotoxin deoxynivalenol (DON) as the carbon source. This is the first study of the genome sequence of the DON-degrading genus Nocardioides, and it facilitates the study of genes encoding the DON-degrading pathway.

## Abstract (precis)

Actinomycete Nocardioides sp. strain LS1, isolated from wheat leaf, is a bacterium that degrades and assimilates the mycotoxin deoxynivalenol (DON) as the carbon source.

## ANNOUNCEMENT

The mycotoxin deoxynivalenol (DON; 3α,7α,15-trihydroxy-12,13-epoxytrichothec-9-en-8-one) is classified as a trichothecene and is commonly accumulated in crop cereals infected by members of the Fusarium graminearum species complex (1, 2). The cereals in which DON accumulates due to F. graminearum infection can contaminate agricultural systems, resulting in harm to both humans and livestock (3 – 5) and leading to large economic losses (6). Since production and storage are threatened by DON contamination (7, 8), a more effective approach for the degradation of DON is required (9, 10).

We have isolated several DON-degrading bacteria classified as Nocardioides spp. and the closely related Marmoricola sp., including strain LS1 (11 – 13). Therefore, this genome data of strain LS1 will be essential information for research and application of DON-degrading bacteria that belong to Nocardioides/Marmoricola. This is the first report of a genome sequence for a DON-degrading bacterium belonging to genus Nocardioides.

Nocardioides sp. strain LS1, isolated from wheat leaves at the National Institute for Agro-Environmental Sciences, Tsukuba, Ibaraki, Japan (13), was cultured onto 3-fold diluted Reasoner’s 2A (R2A) agar (Wako, Tokyo, Japan) at 28°C for 7 days, and a single colony was picked for sequencing. The genomic DNA was extracted using a Wizard Genomic DNA purification kit (Promega, USA). Paired-end DNA libraries were prepared using the Nextera XT kit (Illumina, USA) for sequencing with the MiSeq platform (Illumina, USA). The sequencing generated a total of 1,444,938 paired-end reads, and the total length of reads was 810,060,185 bases. The average length of reads was 561 bases. The raw sequencing reads were quality trimmed using the FASTQ preprocessing program fastp (14) with default parameter settings. *De novo* assembly was performed using the SPAdes genome assembler version 3.12.0 (15) with three options, “-k auto,” “-careful,” and “-cov-cutoff 10.0,” which obtained 16 contigs. These contigs were connected into eight scaffolds by PCR and subsequent Sanger sequencing analysis. The scaffolds, produced from the draft genome of Nocardioides sp. strain LS1, consisted of 4,536,969 bases with a G+C content of 71.31%. The 16S rRNA gene sequence of strain LS1, based on a BLASTn search with default parameter settings and an E value of 1.0E−10, showed 100% identity to that of Nocardioides ginsengisegetis strain Gsoil 485 (16). Average nucleotide identity (ANI) analysis using the online ANI calculator (http://enve-omics.ce.gatech.edu/ani/index) revealed a two-way ANI value of 80.80% with Nocardioides sp. strain 603 and a value of 80.70% with Nocardioides sp. strain JS614. Genome completeness and contamination were estimated using CheckM (17). The final draft genome was 97.92% complete. Annotation of the draft genome was performed using the DFAST version 1.0.0 pipeline (18), and 4,431 putative coding sequences were indicated.

The genes for previously characterized DON-degrading enzymes, including DdnA (19) and DepA (20), were reported. Although strain LS1 has the capacity to both degrade and assimilate DON (13), the DFAST genome annotation indicated an absence of the genes that encode DON-degrading enzymes. Thus, the results implied the strain LS1 has a novel DON-degrading pathway.

### Data availability.

The draft genome sequence was deposited at DDBJ/EMBL/GenBank under accession numbers BIFF01000001 to BIFF01000008. Raw sequencing data were deposited in the DDBJ SRA database under BioProject number PRJDB7627 and BioSample number SAMD00151336.
